# The timing of arterial thrombectomy on neurological recovery in patients with acute ischemic stroke: A retrospective observational study

**DOI:** 10.1097/MD.0000000000047634

**Published:** 2026-02-20

**Authors:** Guiguo Yan, Weihai Li, Baihai Guo, Shibin Wen, Rui Zhang, Lijuan Yin, Fei Wang, Peng Liang

**Affiliations:** a Department of Neurology, Shanghai General Hospital, Jiuquan Hospital, Jiuquan, China.

**Keywords:** acute ischemic stroke, arterial thrombectomy, complication, intravenous thrombolysis, neurological function

## Abstract

Arterial thrombectomy (AT) is a cornerstone in the treatment of acute ischemic stroke (AIS) due to large vessel occlusion. However, the optimal therapeutic time window and the best management strategy for patients presenting beyond the conventional 4.5-hour timeframe remain areas of active investigation and debate. This retrospective cohort study aimed to analyze the effect of timing of AT on recovery in AIS. We retrospectively analyzed 117 AIS patients admitted between January 2021 and January 2023. Participants were categorized into 3 groups: early AT (onset-to-AT < 4.5 hours), late AT (onset-to-AT ≥ 4.5 hours), and late AT + intravenous thrombolysis (IT). Outcomes compared included clinical efficacy, National Institutes of Health Stroke Scale (NIHSS) scores, serum levels of neurotrophic factors, brain-derived neurotrophic factor, vascular endothelial growth factor, residual stenosis, vessel reocclusion, 3-month mortality, and 1-month complications. The total effective rate was higher in the early AT and late AT + IT groups than in the late AT group. Pretreatment NIHSS scores and serum neurological marker levels were comparable across all groups. After treatment, the early AT and late AT + IT groups showed significantly lower NIHSS scores, higher serum levels of neurological markers, and improved treatment efficiency compared to the late AT group. Prognosis-related markers also indicated better outcomes in these 2 groups. Additionally, complications such as mucocutaneous ecchymosis, gastrointestinal bleeding, and intracranial bleeding were significantly reduced in the early AT and late AT + IT groups. AT within 4.5 hours of stroke onset improves efficacy, reduces neurological injury, and decreases complications. For patients presenting beyond 4.5 hours, combining AT with IT achieves comparable therapeutic benefits.

## 1. Introduction

Acute ischemic stroke (AIS) is a common clinical cerebrovascular disease, accounting for 60% to 80% of all strokes according to relevant investigations.^[1]^ It has a high incidence rate and a high disability and mortality rate. Currently, AIS is believed to be caused by thrombus blocking the blood vessels and leading to pathological reactions. The main symptoms include dizziness, headache, facial asymmetry, limb numbness, and consciousness disorders, which directly threaten the patient’s life and reduce their quality of life.^[2,3]^ Therefore, timely and effective treatment is of great significance for AIS patients. The main goal of clinical treatment for AIS is to promote the restoration of blood flow in the ischemic area and avoid oxygen deprivation and neuronal damage. Studies have showed that low concentration of brain-derived neurotrophic factor (BDNF) in AIS was a factor in poor prognosis in terms of functional status of patients.^[4]^ BDNF protected neurons from apoptosis, enhanced neuroplasticity and improved cerebral blood flow.^[5]^ Another neurotrophic factor (NTF) vascular endothelial growth factor (VEGF) was upregulated in the ischemic brain and was considered interesting potential treatment strategies in AIS.^[6–8]^ In stroke mice, atorvastatin promoted brain plasticity by regulating BDNF and VEGF.^[9]^ Therefore, NTFs play an important role in AIS and can help us better understand the pathogenesis of AIS.

Treatment options include intravenous or arterial thrombectomy (AT) and AT. However, intravenous or AT has limitations such as poor efficacy and more complications, which restrict its clinical application.^[10,11]^ With the advancement of interventional treatment techniques in recent years, the application of AT has gradually expanded in clinical practice. Studies have shown that it can open blocked blood vessels in the short term, save ischemic penumbra, and has a high safety profile, especially for AIS patients.^[12]^ AT for AIS patients has strict time dependence. Some scholars believe that early AT has better short-term and long-term efficacy,^[13]^ while others believe that the duration of AT can be reasonably extended.^[14]^ There is still some controversy regarding the specific timing of AT for AIS patients in current clinical practice. Therefore, to contribute to this ongoing debate and to evaluate the potential of combination therapy for late-presenting patients, this study retrospectively analyzed the clinical data of 117 AIS patients admitted to our hospital. We aimed to compare not only the efficacy and safety of AT within versus beyond the 4.5-hour time window but also to assess the value of bridging intravenous thrombolysis (IT) in the late-presenting cohort. Our investigation encompassed clinical outcomes, neurological function recovery (as measured by National Institutes of Health Stroke Scale [NIHSS] and serum neurotrophic factors), and key prognostic and safety indicators.

## 2. Methods

### 2.1. Study design and population

**Figure 1.**
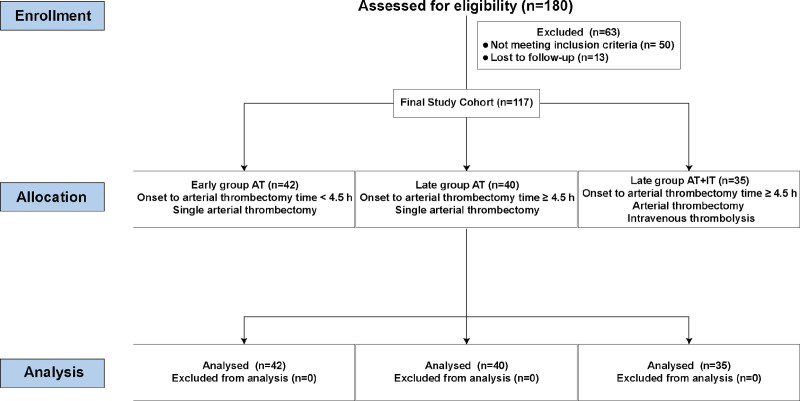
The flowchart of patient selection process.

A retrospective analysis of the clinical data of 117 AIS patients admitted to our hospital from January 2021 to January 2023 was conducted. Based on the timing of AT, the patients were divided into an early group (onset-to-AT time < 4.5 hours, n = 42, single AT), a late group (onset-to-AT time ≥ 4.5 hours, n = 40, single AT) and a late group which accept combination treatment (onset-to-AT time ≥ 4.5 hours, n = 35, AT + IT). It was worth noting revascularization time was the time endpoint. The basic information and clinical data, including gender, age, body mass index, onset time, and underlying diseases, were collected for all 3 groups. *Inclusion criteria:* Age between 40 and 80 years, regardless of gender. Clinical diagnosis of AIS according to the clinical diagnostic criteria,^[15]^ including acute onset, focal neurological deficits, imaging findings of responsible lesions, and signs and symptoms lasting >24 hours; exclusion of nonvascular etiology; exclusion of cerebral hemorrhage by brain computed tomography (CT) or magnetic resonance imaging. Confirmed anterior circulation large vessel occlusion on computed tomography angiography or magnetic resonance angiography, eligible for endovascular treatment. First-ever stroke. Meeting the indications for AT. Complete clinical data. *Exclusion criteria:* Severe underlying diseases that could confound outcomes, such as uncontrolled hyperlipidemia, hyperglycemia, or hypertension; Pre-stroke modified Rankin scale score >2, indicating significant preexisting disability; Cerebral hemorrhage, intracranial tumor, or previous cerebrovascular stenting surgery; Cognitive impairment or psychiatric disorders that would preclude accurate neurological assessment; Estimated survival time <1 year due to malignancy or other severe comorbidities; Severe renal or hepatic insufficiency (e.g., serum creatinine > 2.0 mg/dL); Pregnancy; Known allergy to contrast media; Poor compliance with follow-up. The patient selection process was summarized in Figure 1. This study was approved by the Ethics Committee of Shanghai General Hospital, Jiuquan Hospital. The procedures were conducted in accordance with the ethical standards set forth by the Committee on Human Experimentation and the Helsinki Declaration of 1964, as revised in 2013. Informed consent was waived by the Ethics Committee of Shanghai General Hospital, Jiuquan Hospital for this retrospective study due to the exclusive use of de-identified patient data, which posed no potential harm or impact on patient care. This waiver was approved by the institutional review board and ethics committee of our institution in accordance with regulatory and ethical guidelines pertaining to retrospective studies.

### 2.2. Preoperative preparation

*Medical history collection:* assess the past medical history, including cerebrovascular diseases, bleeding disorders, surgical history, drug use history, etc. Confirm whether there are any contraindications for AT (such as severe coagulation disorders, extensive cerebral infarction); According to the NIHSS, evaluate the severity of stroke and check the state of consciousness, language function, body movement, sensory function, etc; *Laboratory tests:* hematological tests to rule out coagulation abnormalities, biochemical tests to clarify basal metabolic conditions, and cardiac assessment to determine whether there is atrial fibrillation or other arrhythmias to determine the potential source of emboli; *Imaging examination:* CT scan to rule out intracranial hemorrhage, determine the size of the infarct core, and CT perfusion imaging to evaluate the ratio of the ischemic penumbra to the infarct core. The Alberta Stroke Program Early CT Score was used to quantify early ischemic changes on non-contrast CT. The site of arterial occlusion including, internal carotid artery, M1, or M2 segment of the middle cerebral artery was confirmed by computed tomography angiography or magnetic resonance angiography; *Symptomatic treatments*: nutritional support, oxygen free radical clearance, platelet aggregation inhibition, dehydration, intracranial pressure reduction, lipid control, blood pressure control, blood glucose control, and maintenance of electrolyte balance.

### 2.3. Intraoperative treatment

All patients underwent AT. The specific procedure was as follows: Local anesthesia was performed with 10 mL of 1.0% lidocaine (Shandong Hualu Pharmaceutical Co., Ltd., National Drug Approval Number: H37022147). After the anesthesia took effect, the modified Seldinger puncture method was used to puncture the femoral artery. An arterial sheath (QiSheng Medical Devices [Shanghai] Co., Ltd., model: 6F) was placed which can support the procedures of most thrombectomy, and the responsible vessel was determined based on the results of angiography. The state of collateral circulation was determined, and a guiding catheter was placed into the responsible artery. Under the guidance of a microcatheter, the microcatheter was advanced to the distal occlusion of the artery. The Solitaire AB stent (Shanghai Kehui Medical Equipment Co., Ltd., model: SAB-6-20, National Medical Device Approval Number: 20173136562) was released. After the stent was released, it was left in place for 3 to 5 minutes. Then, the stent and microcatheter were withdrawn together, and the thrombus retrieved by the stent was examined. Multiple retrievals could be performed if necessary. The procedure was considered successful if the vessel was successfully recanalized according to angiography and the blood flow in the occluded vessel was satisfactory. Specifically, we considered at least 50% of the infarct area was reperfused, that is TICI score ≥2b is defined as satisfactory revascularization. Here, we didn’t use aspiration because existing cases can meet clinical treatment needs by only using stent extractors, which can quickly and effectively remove thrombus. Moreover, aspiration may increase the risk of damage to the inner wall of the blood vessel. Hemostasis was achieved using a closure device, and the incision was sutured. The early group had an onset-to-AT time <4.5 hours, and the late groups had an onset-to-AT time ≥4.5 hours. For patients in the late group AT + IT, IT was initiated immediately upon diagnosis and confirmation of eligibility, prior to AT. Recombinant tissue plasminogen activator (rtPA, alteplase) was administered at a standard dose of 0.9 mg/kg (maximum 90 mg), with 10% of the total dose given as an intravenous bolus over 1 minute, followed by the remaining 90% infused continuously over 60 minutes. AT was subsequently performed if no significant clinical improvement was observed or as a bridging therapy protocol.

### 2.4. Postoperative management

All patients received intensive monitoring and routine antiplatelet therapy after the procedure particularly neurological monitoring. Continuous monitoring of vital signs (blood pressure, heart rate, oxygen saturation, etc), neurological status, and bleeding risk was required. The options included enteric-coated aspirin tablets (Bayer HealthCare Co., Ltd., 100 mg × 30 tablets, National Drug Approval Number J20171021) at a daily oral dose of 100 mg or clopidogrel hydrogen sulfate tablets (Lepu Pharmaceutical Co., Ltd., 25 mg × 10 tablets, National Drug Approval Number H20123115) at a daily oral dose of 75 mg.

### 2.5. Outcome measures

The clinical outcomes of the 3 groups were observed, including the NIHSS scores and the levels of serum NTF, BDNF, and VEGF before treatment and at 3 weeks after treatment. The residual stenosis, secondary vessel occlusion, and mortality rate at 3 months after treatment were recorded, and the complications within 1 month after the procedure were also documented.

1. *Clinical outcomes:* The clinical outcomes at 3 weeks after treatment were collected for these 3 groups. The outcomes were determined based on the NIHSS scores and symptom changes; *Cure:* NIHSS score decreased by ≥90% compared to before treatment, and symptoms were almost absent; *Significant improvement:* NIHSS score decreased by 45% to 89% compared to before treatment, and symptoms showed a significant improvement trend, with a significant increase in limb muscle strength; *Effective:* NIHSS score decreased by 18% to 44% compared to before treatment, and symptoms were basically under control, with a certain increase in limb muscle strength; *Ineffective:* did not meet the above criteria.^[16]^ Overall response rate = the number of significant improvement and improvement cases/total number × 100%.
2. *Neurological function:* The NIHSS scores before treatment and at 3 weeks after treatment were collected for 3 groups. The scale includes 11 scoring criteria: level of consciousness, gaze, visual field, facial palsy, upper limb motor function, lower limb motor function, limb ataxia, sensation, language, dysarthria, and neglect. The total score ranges from 0 to 42, and the score is positively correlated with the degree of neurological impairment.^[17]^
3. *Serum markers:* Serum markers were collected before treatment and at 3 weeks after treatment. Three milliliters of fasting venous blood from the elbow vein were collected and centrifuged at 1000 rpm for 10 minutes. The supernatant was used for the measurement of NTF, BDNF, and VEGF levels using enzyme-linked immunosorbent assay. Each marker was measured 3 times, and the average of the 3 results was taken as the final result.
4. *Prognosis:* The residual stenosis, secondary vessel occlusion, and mortality rate at 3 months after treatment were recorded for 3 groups.
5. *Complications:* The proportion of patients with skin and mucosal ecchymosis, gastrointestinal bleeding, and intracranial bleeding within 1 month after the procedure was recorded for 3 groups.

### 2.6. Statistical analysis

The data were analyzed using SPSS 25.0 statistical software (IBM Corp., Armonk). Count data were presented as n (%) and analyzed using the chi-square test. Measurement data were checked for normality using the Shapiro–Wilk test. Normally distributed data were reported as mean ± standard deviation and analyzed using one-way ANOVA. Non-normally distributed data were expressed as median (interquartile range; P25, P75) and analyzed using nonparametric tests. *P* < .05 was considered statistically significant. Additionally, a post hoc power analysis was conducted using G*Power software (version 3.1.9.7; Heinrich Heine University Düsseldorf, Düsseldorf, Germany) for the chi-square test of the primary clinical outcome (overall response rate) between the late group AT and the late group AT + IT. Given the observed effect size (w = 0.45), an alpha level of 0.05, and sample sizes of 40 and 35, the achieved power was calculated to be 84.5%, which is above the conventional threshold of 80%.

## 3. Results

### 3.1. Baseline characteristics

There were no statistically significant differences in gender, age, body mass index, onset time, and underlying diseases among the 3 groups (*P* > .05) (Table 1), suggesting that the groups were comparable.

**Table 1:** Comparison of baseline characteristics among the 3 groups.

Variable	Early group AT (n = 42)	Late group AT (n = 40)	Late group AT + IT (n = 35)	*F/χ* ^2^	*P* value
Demographics					
Gender					
Male	24 (57.14%)	25 (62.5%)	24 (68.57%)	1.063	.588
Female	18 (42.86%)	15 (37.5%)	11 (31.43%)		
Age (yr)	55.26 ± 4.29	54.78 ± 4.39	55.26 ± 4.08	0.1702	.844
BMI (kg/m^2^)	27.90 ± 4.32	28.35 ± 4.40	27.52 ± 3.00	0.403	.669
Clinical characteristics					
Onset time (h)	4.50 ± 1.52	5.03 ± 3.12	5.00 ± 2.00	0.669	.514
Baseline NIHSS	16 (14–19)	17 (15–20)	16 (13–19)	1.245	.537
Systolic BP (mm Hg)	148.5 ± 18.2	152.1 ± 20.5	149.8 ± 17.8	0.421	.657
Diastolic BP (mm Hg)	85.2 ± 11.5	87.6 ± 12.8	84.9 ± 10.3	0.659	.519
Blood glucose (mmol/L)	7.8 ± 1.9	8.1 ± 2.2	7.9 ± 2.0	0.231	.794
Atrial fibrillation	5 (11.90)	6 (15.00)	4 (11.43)	0.287	.866
Imaging characteristics					
ASPECTS	8 (7–9)	8 (6–9)	8 (7–9)	0.883	.643
Site of occlusion					
ICA	8 (19.05)	7 (17.50)	6 (17.14)	0.721	.948
MCA-M1	25 (59.52)	26 (65.00)	22 (62.86)		
MCA-M2	9 (21.43)	7 (17.50)	7 (20.00)		
Medical history					
Hypertension	8 (19.05%)	7 (17.50%)	6 (17.14%)	0.9134	.9226
Diabetes	6 (14.29%)	4 (10.00%)	6 (17.14%)		
None	28 (66.67%)	29 (72.50%)	23 (65.72%)		

AT = arterial thrombectomy, ASPECTS = Alberta Stroke Program Early CT Score, BMI = body mass index, ICA = internal carotid artery, IT = intravenous thrombolysis, MCA = middle cerebral artery, National Institutes of Health Stroke Scale.

### 3.2. Clinical outcome

We compared the clinical outcomes of the early treatment group AT (n = 42), the late treatment group AT (n = 40) and the late treatment group AT + IT (n = 35) (Table 2). The results are as follows: the cure rate in the early group AT was 47.62% (20 patients), 25.00% (10 patients) in the late group AT and 40.00% (14 patients) in the late group AT + IT. Similarly, significant improvement was observed in 35.71% (15 patients) of the early group AT, 30.00% (12 patients) in the late group AT and 51.43% (14 patients) in the late group AT + IT. The effective rates were 9.52% (4 patients) for the early group AT, 27.5% (11 patients) for the late group AT, and 8.57% (3 patients) for the late group AT + IT, while the ineffective rate was much lower in the early group AT at 7.14% (3 patients) compared to 17.50% (7 patients) in the late group AT. There were no ineffective patients in the late group AT + IT. Overall, the response rate (cured + significant improvement + effective) was 92.86% in the early group AT, 82.5% in the late group AT and 100% in the late group AT + IT. Statistical analysis showed a significant difference in the overall response rate among the 3 groups (*χ*^2^ = 18.44, *P* = .018), indicating that the early treatment group achieved significantly better outcomes compared to the late group. What’s more, although the treatment time was in advanced stage, AT combined with IT treatment can still achieve good therapeutic effects.

**Table 2:** Comparison of clinical outcomes among the 3 groups.

Situation	Early group AT (n = 42)	Late group AT (n = 40)	Late group AT + IT (n = 35)	*χ* ^2^	*P* value
Cured	20 (47.62%)	10 (25.00%)	14 (40.00%)		
Significant improvement	15 (35.71%)	12 (30.00%)	18 (51.43%)		
Effective	4 (9.52%)	11 (27.50%)	3 (8.57%)		
Ineffective	3 (7.14%)	7 (17.50%)	0 (0.00%)		
Overall response rate	39 (92.86%)	33 (82.50%)	35 (100.00%)	18.44	.018

AT = arterial thrombectomy, IT = intravenous thrombolysis.

### 3.3. NIHSS scores and neurological injury serum markers

**Figure 2.**
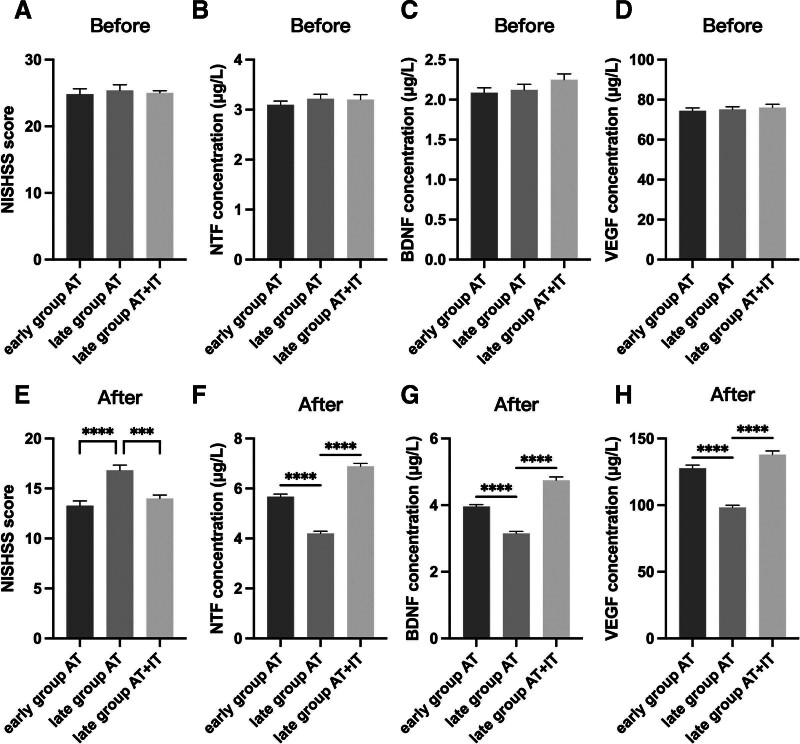
Comparison of NIHSS scores and neurological injury serum markers before and after treatment among the 3 groups. (A) NIHSS scores before treatment of the patients. (B–D) Neurological injury serum markers 3 weeks after treatment of the patients. (E) NIHSS scores 3 weeks after treatment of the patients. (F–H) Neurological injury serum markers 3 weeks after treatment of the patients. ***P < .0001, ****P < .0001. The statistical method was one-way ANOVA.

There were no statistically significant differences in NIHSS scores and serum levels of NTF, BDNF, and VEGF among the 3 groups prior to treatment (*P* > .05). However, after treatment, it was observed that the NIHSS score was lower and the serum levels of NTF, BDNF, and VEGF were higher in the early group AT and the late group AT + IT compared to the late group AT (*P* < .05) (Fig. 2). These findings suggest that initiating AT within 4.5 hours of onset could potentially reduce neurological damage in patients with acute ischemic stroke. When the onset time was more than 4.5 hours, AT combined with IT treatment was more effective than AT alone.

### 3.4. Prognostic indicators and complications

We compared prognostic indicators and complications among the 3 groups (Table 3). We found that, for prognostic indicators, significant differences were observed. The late group AT exhibited the highest rates of residual stenosis (22.5%) and secondary occlusion of blood vessels (20%). In contrast, the early group AT showed much lower rates for both (4.76% and 2.38%, respectively) and the rates in the late group AT + IT were 11.43% and 5.71%, respectively. While the incidence of death was low across all groups, the late group AT had a slightly higher rate (5%) compared to the early group AT (2.38%) and the late group AT + IT (2.86%). Overall, the late group AT had the highest total of prognostic issues (47.5%), followed by the late group AT + IT (20%) and the early group AT (9.52%). The *P*-value for prognostic indicators (.0003) indicates a statistically significant difference among the 3 groups. In terms of complications, the late group AT also had the highest rates, with skin and mucosal ecchymosis (10%), gastrointestinal bleeding (7.5%) and intracranial bleeding (5.00%). The early group AT and late group AT + IT had lower complication rates. Especially, no intracranial bleeding was observed in the 2 groups. The total complication rate was highest in the late group AT (22.5%), compared to the early group AT (4.76%) and late group AT + IT (8.56%). The *P*-value for complications (.0357) also suggested a significant difference among the groups. In conclusion, the late group AT had the highest rates of both prognostic issues and complications, with statistically significant differences compared to the other groups, suggesting that early intervention may reduce the risk of adverse outcomes in patients with AIS. Furthermore, AT combined with IT treatment can also show better effects compared with AT treatment alone, so combined treatment may be an effective option for advanced patients.

**Table 3:** Comparison of prognostic indicators and complications among the 3 groups.

Variable	Early group AT (n = 42)	Late group AT (n = 40)	Late group AT + IT (n = 35)	*χ* ^2^	*P* value
Prognostic indicators					
Residual stenosis	2 (4.76%)	9 (22.50%)	4 (11.43%)		
Secondary occlusion of blood vessels	1 (2.38%)	8 (20.00%)	2 (5.71%)		
Death	1 (2.38%)	2 (5.00%)	1 (2.86%)		
Total	4 (9.52%)	19 (47.50%)	7 (20.00%)	16.33	.0003
Complications					
Skin and mucosal ecchymosis	1 (2.38%)	4 (10.00%)	2 (5.71%)		
Gastrointestinal bleeding	1 (2.38%)	3 (7.50%)	1 (2.86%)		
Intracranial bleeding	0 (0.00%)	2 (5.00%)	0 (0.00%)		
Total	2 (4.76%)	9 (22.50%)	4 (11.43%)	6.666	.0357

AT = arterial thrombectomy, IT = intravenous thrombolysis.

## 4. Discussion

AIS is a clinically common disease, especially in the middle-aged and elderly population. With the increasing aging population, the incidence of AIS is gradually increasing and has received high clinical attention.^[18]^ The exact mechanism of this disease is still not fully understood, but it is believed to be caused by blockage and interruption of cerebral blood vessels due to various factors, leading to ischemia and brain damage.^[19,20]^ The fundamental cause of the disease is the blockage of cerebral arteries by blood clots, and the duration of blockage is directly proportional to the risk of death.^[21]^ Therefore, timely and effective treatment measures to promote reperfusion of occluded blood vessels are of great significance in saving patients’ lives. Many patients do not receive timely treatment after AIS onset due to resource constraints such as limited medical resources and inconvenient transportation. This is the case with the late group patients in our study. AT and endovascular thrombectomy are common treatment measures for AIS patients, with the former having limitations such as limited scope of action and unsatisfactory long-term efficacy.^[22]^ Endovascular thrombectomy is a new type of interventional thrombectomy technique developed in recent years. It involves using a vacuum to extract the thrombus near the responsible artery or pulling it into the proximal catheter position after contact with the thrombus in order to successfully perform endovascular thrombectomy, assisting patients in promoting blood flow restoration and reducing neurological damage.^[23]^ According to relevant studies,^[24]^ endovascular thrombectomy can significantly reduce the occurrence of secondary thrombosis, allowing AIS patients to achieve better prognosis and improve their quality of life. However, there is still no consensus in the clinical community on the specific timing of endovascular thrombectomy to improve patient outcomes. This study found that the overall effective rate in the early group AT was higher than that in the late group AT (*P* < .05), suggesting that endovascular thrombectomy performed within 4.5 hours of onset is more effective for AIS patients. This is consistent with the findings of Pandhi et al’s study^[25]^ that early thrombectomy improves outcomes in AIS patients. Early thrombectomy can open the blood vessels in a timely manner, salvage the ischemic penumbra, and expand the balloon or release the stent when there is a risk of secondary occlusion due to vessel stenosis or dissection, thus restoring the blood vessels to a good condition and achieving the effectiveness of emergency treatment, increasing the chances of patients recovering to their pre-onset state and achieving desired outcomes. In our study, we also provided evidence of the effectiveness of combined AT and IT treatment in the late group. Although previous studies have analyzed the effect of mechanical thrombectomy in patients with AIS more than 6 hours after onset, indicating that mechanical thrombectomy was a recommended option for patients with a large ischemic penumbra but not complete infraction,^[26]^ our results demonstrated that the effect of AT combined with IT in the late group was superior to that of AT alone.

AIS patients already have varying degrees of neurological damage before treatment, mainly manifested as an increase in NIHSS score and changes in various neuroinjury biomarker levels. NTF, BDNF, and VEGF are important cell protective factors for nerve cells, among which NTF and BDNF can accelerate nerve cell regeneration and promote axonal growth, thus improving nerve function^[27]^; VEGF is a peptide growth factor that can accelerate the division of endothelial cells, thereby promoting angiogenesis, facilitating collateral circulation in the infarct area, and helping to reestablish nerve function.^[28]^ Deng et al found that early thrombectomy treatment can better improve neurological function in AIS patients.^[29]^ The results of this study showed that the NIHSS score after treatment in the early group AT and late group AT + IT were lower than that in the late group and the serum levels of NTF, BDNF, and VEGF were higher than those in the late group AT, indicating that endovascular thrombectomy performed within 4.5 hours of onset can reduce neurological damage in AIS patients and combination treatment was more effective than single AT treatment, which is consistent through the blood vessels to the site of occlusion and remove the thrombus, promptly restoring the cerebral blood flow status in the infarct area and relieving neurological damage caused by cerebral ischemia and hypoxia. FLADT J et al also found that early thrombectomy can alleviate neurological damage in AIS patients,^[30]^ further confirming the findings of this study. The time from onset to surgery directly affects the effectiveness and prognosis of AIS patients. Mierzwa et al^[31]^ advocated early endovascular thrombectomy for AIS patients after onset to reduce the occurrence of complications and improve patient prognosis. The results of this study showed that the residual stenosis and secondary occlusion rates in the early group AT were lower than those in the late group AT, and the rates of skin mucosal ecchymosis and complications such as gastrointestinal or intracranial hemorrhage were lower than those in the late group AT, suggesting that endovascular thrombectomy performed within 4.5 hours of onset can improve the prognosis of AIS patients and reduce the occurrence of complications. Early endovascular thrombectomy can better promote the reestablishment of neurological function, accelerate the release of neuroprotective factors, reduce the release of neural-damaging factors, alleviate neuronal damage, reduce apoptosis of brain neurons, and provide good protection for cognitive function, reducing brain tissue necrosis and bleeding, and improving patient prognosis. In addition, early endovascular thrombectomy can also reduce the dose and duration of postoperative antiplatelet use, thereby reducing the incidence of unnecessary complications such as bleeding. What’s more, although it is more difficult to treat advanced patients, the AT combined with IT regimen has shown better results in improving prognosis and reducing complications compared with AT treatment alone. Therefore, combined treatment may be an effective option for advanced patients.

Nevertheless, the present study is not without its limitations. First, the sample size, particularly for the late group receiving combination therapy was relatively modest. Although our post hoc analysis indicated sufficient power for the primary outcomes, the small sample size may still limit the generalizability of our findings and the ability to perform more extensive subgroup analyses. Future research with a larger, multicenter cohort is warranted to validate our results. Second, this study used a retrospective cohort study design, which cannot completely eliminate potential confounding factors and information bias, but we collected as much information as possible to show that the 3 groups were comparable. Third, imaging indicators and recurrence rates were not statistically analyzed, but we tried our best to collect other indicators. Despite these limitations, this support for determining the optimal timing of AT in AIS patients, and this treatment approach is expected to be applied in actual clinical practice to improve short-term efficacy and long-term prognosis in AIS patients. Future research can address these limitations by using more refined prospective designs, extending the study period, and conducting multicenter studies with larger sample sizes.

## 5. Conclusion

In conclusion, compared with AT performed ≥4.5 hours after onset, AT performed <4.5 hours after onset can improve the effectiveness of AIS patients, alleviate neurological damage, reduce the occurrence of complications, and improve prognosis. When the onset time of AIS patients was more than 4.5 hours, AT and IT combination treatment can also achieve the comparable therapeutic effects.

## Acknowledgments

The authors express their appreciation to staff in Shanghai General Hospital, Jiuquan Hospital, for their technical assistance.

## Author contributions

**Conceptualization:** Guiguo Yan, Shibin Wen, Peng Liang.

**Data curation:** Guiguo Yan, Weihai Li, Baihai Guo, Rui Zhang, Lijuan Yin, Fei Wang.

**Investigation:** Weihai Li, Baihai Guo, Shibin Wen, Rui Zhang, Lijuan Yin, Fei Wang, Peng Liang.

**Writing – original draft:** Guiguo Yan, Weihai Li, Baihai Guo, Shibin Wen, Rui Zhang, Lijuan Yin, Fei Wang.

**Writing – review & editing:** Guiguo Yan, Peng Liang.

## References

[1] Kamel H Parikh NS Chatterjee A. Access to mechanical thrombectomy for ischemic stroke in the United States. Stroke. 2021;52:2554–61. 33980045 10.1161/STROKEAHA.120.033485 PMC8316281

[2] Fladt J d’Esterre CD Joundi R McDougall C Gensicke H Barber P. Acute stroke imaging selection for mechanical thrombectomy in the extended time window: is it time to go back to basics? A review of current evidence. J Neurol Neurosurg Psychiatry. 2022;93:238–45. 35115388 10.1136/jnnp-2021-328000

[3] Bhatia KD Chowdhury S Andrews I. Association between thrombectomy and functional outcomes in pediatric patients with acute ischemic stroke from large vessel occlusion. JAMA Neurol. 2023;80:910–8. 37486670 10.1001/jamaneurol.2023.2303 PMC10366944

[4] Lasek-Bal A Jędrzejowska-Szypułka H Różycka J. Low concentration of BDNF in the acute phase of ischemic stroke as a factor in poor prognosis in terms of functional status of patients. Med Sci Monit. 2015;21:3900–5. 26656843 10.12659/MSM.895358 PMC4684138

[5] Chan A Yan J Csurhes P Greer J McCombe P. Circulating brain derived neurotrophic factor (BDNF) and frequency of BDNF positive T cells in peripheral blood in human ischemic stroke: effect on outcome. J Neuroimmunol. 2015;286:42–7. 26298323 10.1016/j.jneuroim.2015.06.013

[6] Góra-Kupilas K Jośko J. The neuroprotective function of vascular endothelial growth factor (VEGF). Folia Neuropathol. 2005;43:31–9. 15827888

[7] Dzietko M Derugin N Wendland MF Vexler ZS Ferriero DM. Delayed VEGF treatment enhances angiogenesis and recovery after neonatal focal rodent stroke. Transl Stroke Res. 2013;4:189–200. 23926451 10.1007/s12975-012-0221-6 PMC3733573

[8] Geiseler SJ Morland C. The Janus Face of VEGF in Stroke. Int J Mol Sci. 2018;19:1362. 29734653 10.3390/ijms19051362 PMC5983623

[9] Chen J Zhang C Jiang H. Atorvastatin induction of VEGF and BDNF promotes brain plasticity after stroke in mice. J Cereb Blood Flow Metab. 2005;25:281–90. 15678129 10.1038/sj.jcbfm.9600034 PMC2804085

[10] Dhillon PS Butt W Podlasek A. Association between time to treatment and clinical outcomes in endovascular thrombectomy beyond 6 hours without advanced imaging selection. J Neurointerv Surg. 2023;15:336–42. 35296526 10.1136/neurintsurg-2021-018564

[11] Hubert GJ Hubert ND Maegerlein C. Association between use of a flying intervention team vs patient interhospital transfer and time to endovascular thrombectomy among patients with acute ischemic stroke in Nonurban Germany. JAMA. 2022;327:1795–805. 35510389 10.1001/jama.2022.5948 PMC9092197

[12] Krishnan R Mays W Elijovich L. Complications of mechanical thrombectomy in acute ischemic stroke. Neurology. 2021;97(20 Suppl 2):S115–25. 34785610 10.1212/WNL.0000000000012803

[13] Mierzwa AT Rao R Al Kasab S. Early and late basilar artery thrombectomy time window outcomes. Front Neurol. 2024;15:1352310. 38343711 10.3389/fneur.2024.1352310 PMC10858612

[14] Deng Q Zhang L Liu Y. Effect of time window on endovascular thrombectomy with or without intravenous thrombolysis in acute ischemic stroke: results from DIRECT-MT. Cerebrovasc Dis. 2024;53:176–83. 37598670 10.1159/000533231

[15] Bendszus M Fiehler J Subtil F. Endovascular thrombectomy for acute ischaemic stroke with established large infarct: multicentre, open-label, randomised trial. Lancet. 2023;402:1753–63. 37837989 10.1016/S0140-6736(23)02032-9

[16] Sarraj A Haessan A Abeaham MG. Endovascular thrombectomy for large ischemic stroke across ischemic injury and penumbra profiles. JAMA. 2024;331:750–63. 38324414 10.1001/jama.2024.0572 PMC10851143

[17] Mitchell PJ Yan B Churilov L. Endovascular thrombectomy versus standard bridging thrombolytic with endovascular thrombectomy within 4–5 h of stroke onset: an open-label, blinded-endpoint, randomised non-inferiority trial. Lancet. 2022;400:116–25. 35810757 10.1016/S0140-6736(22)00564-5

[18] Donato AA Goswami V. In acute ischemic stroke, thrombectomy was not noninferior to alteplase + thrombectomy for 90-d functional independence. Ann Intern Med. 2022;175:JC125. 36315954 10.7326/J22-0088

[19] Jadhav AP Desai SM Jovin TG. Indications for mechanical thrombectomy for acute ischemic stroke: current guidelines and beyond. Neurology. 2021;97(20 Suppl 2):S126–36. 34785611 10.1212/WNL.0000000000012801

[20] Consoli A Gory B. Long-term results of mechanical thrombectomy for large ischaemic stroke. Lancet. 2024;403:700–1. 38346443 10.1016/S0140-6736(24)00158-2

[21] Aroor SR Asif KS Potter-Vig J. Mechanical thrombectomy access for all? Challenges in increasing endovascular treatment for acute ischemic stroke in the United States. J Stroke. 2022;24:41–8. 35135058 10.5853/jos.2021.03909 PMC8829477

[22] Sheth SA. Mechanical thrombectomy for acute ischemic stroke. Continuum (Minneap Minn). 2023;29:443–61. 37039404 10.1212/CON.0000000000001243

[23] Aboul-Nour H Maraey A Jumah A. Mechanical thrombectomy for acute ischemic stroke in metastatic cancer patients: a nationwide cross-sectional analysis. J Stroke. 2023;25:119–25. 36592967 10.5853/jos.2022.02334 PMC9911847

[24] Pandhi A Chandra R Abdulrazzak MA. Mechanical thrombectomy for acute large vessel occlusion stroke beyond 24 h. J Neurol Sci. 2023;447:120594. 36893513 10.1016/j.jns.2023.120594

[25] Long B Gottlieb M. Mechanical thrombectomy for anterior circulation stroke beyond 6 h from stroke onset. Acad Emerg Med. 2022;29:801–3. 35490342 10.1111/acem.14516

[26] Nogueira RG Jadhav AP Haussen DC. Thrombectomy 6 to 24 hours after stroke with a mismatch between deficit and infarct. N Engl J Med. 2018;378:11–21. 29129157 10.1056/NEJMoa1706442

[27] Naldi A Pracucci G Cavallo R. Mechanical thrombectomy for in-hospital stroke: data from the Italian registry of endovascular treatment in acute stroke. J Neurointerv Surg. 2023;15:e426–32. 36882319 10.1136/jnis-2022-019939

[28] Li Q Abdalkader M Siegler JE. Mechanical thrombectomy for large ischemic stroke: a systematic review and meta-analysis. Neurology. 2023;101:e922–32. 37277200 10.1212/WNL.0000000000207536 PMC10501098

[29] Gentile L Pracucci G Saia V. Mechanical thrombectomy in patients with heart failure: the Italian registry of endovascular treatment in acute stroke. Neurol Sci. 2023;44:3577–85. 37199875 10.1007/s10072-023-06830-9

[30] Nicolini E Saia V Lorenzano S. Mechanical thrombectomy in young patients with large vessel occlusion-related ischemic stroke: data from the Italian Registry of endovascular treatment in acute stroke. Eur J Neurol. 2023;30:3751–60. 37565375 10.1111/ene.16035

[31] Jovin TG Nogueira RG Lansberg MG. Thrombectomy for anterior circulation stroke beyond 6 h from time last known well (AURORA): a systematic review and individual patient data meta-analysis. Lancet. 2022;399:249–58. 34774198 10.1016/S0140-6736(21)01341-6

